# The phase-I study of a therapeutic vaccine to ATL patients with autologous dendritic cells pulsed with peptides corresponding to Tax-specific CTL epitopes

**DOI:** 10.1186/1742-4690-11-S1-O2

**Published:** 2014-01-07

**Authors:** Youko Suehiro, Atsuhiko Hasegawa, Tadafumi Iino, Amane Sasada, Nobukazu Watanabe, Ilseung Choi, Tetsuya Fukuda, Shigeo Takaishi, Ryuji Tanosaki, Atae Utsunomiya, Osamu Miura, Masao Matsuoka, Takanori Teshima, Koichi Akashi, Jun Okamura, Mari Kannagi, Naokuni Uike

**Affiliations:** 1 Department of Hematology, National Kyushu Cancer Center, Fukuoka, Japan; 2 Department of Immunotherapeutics, Tokyo Medical and Dental University, Tokyo, Japan; 3 Department of Medicine and Biosystemic Science, Kyushu University, Fukuoka, Japan; 4 Institute of Medical Science, University of Tokyo, Tokyo, Japan; 5 Department of Hematology, Tokyo Medical and Dental University, Tokyo, Japan; 6 Center for Advanced Medicine Innovation, Kyushu University, Fukuoka, Japan; 7 Clinical Laboratory Division, National Cancer Center Hospital, Tokyo, Japan; 8 Department of Hematology, Imamura Bun-in Hospital, Kagoshima, Japan; 9 Institute for Virus Research, Kyoto University, Kyoto, Japan; 10 Department of Hematology, Hokkaido University, Hokkaido, Japan; 11 Institute for Clinical Research, National Kyushu Cancer Center, Fukuoka, Japan

## 

In the last decade, various kinds of clinical trials of hematopoietic stem cell transplantation (HSCT) have been performed for ATL patients, and showed therapeutic effects with long-term remission in a part of recipients. Our previous finding of activation of CD8+ Tax-specific cytotoxic T-lymphocytes (CTL) in post-HSCT ATL patients indicated the presence of Tax expression in vivo and potential contributions of CTL to GVL effects. These findings let us attempt developing an anti-ATL therapeutic vaccine consisting of autologous dendritic cells (DC) pulsed with Tax peptides corresponding to the major epitopes of Tax-specific CTL previously identified from HLA-A2, A24 or A11-possessing post-HSCT ATL patients. In preliminary experiments, the DC induced with a conventional method showed matured phenotype and produced IL-12 in 2 of 3 ATL patients tested. Under the official approval by the institutional ethical committees, we conducted the phase-I clinical study of anti-ATL immunotherapy for ATL patients at stable conditions after other therapy. The peptide-pulsed DC was subcutaneously injected for three times with 2 weeks intervals. The first patient showed a significant reduction in the proviral loads in 1 week after administration of DC, which gradually increase during the treatment, then decreased again at 8 weeks after the initiation of the therapy. Reduction in the size of surface lymph nodes by CT confirmed the therapeutic effects. So far two patients completed the course of the study without severe side effects except for fever and skin rash, and their clinical outcomes were partial remission and stable disease respectively.

